# A Pacemaker that was Avoided

**DOI:** 10.7759/cureus.2555

**Published:** 2018-04-30

**Authors:** Husnain Waseem, Arsalan Talib Hashmi, Maham Anser, Neehal Wali, Daniel Rodriguez, Yisachar Greenberg

**Affiliations:** 1 Internal Medicine, Maimonides Medical Center, New York, USA; 2 Internal Medicine, Sir Ganga Ram Hospital, Lahore, PAK; 3 Cardiology, Maimonides Medical Center, New York, USA

**Keywords:** hypothyroidism, heart block, pace maker

## Abstract

Thyroxine is an essential hormone in the human body and exerts many effects on the cardiovascular system. The low metabolic state in hypothyroidism causes bradycardia and reduced cardiac contractility leading to reduced cardiac output. Severe bradycardia and atrioventricular (AV) blocks secondary to hypothyroidism have also been reported. We present a case of severe hypothyroidism causing a high-grade AV block which was successfully treated with thyroxine hormone replacement without requiring cardiac pacemaker placement.

## Introduction

Thyroxine is an essential hormone in the human body and exerts many effects on the cardiovascular system. The low metabolic state in hypothyroidism causes bradycardia and reduced cardiac contractility leading to reduced cardiac output. Most of the cardiovascular effects of hypothyroidism are reversible with thyroid hormone replacement therapy [1]. Severe bradycardia and atrioventricular (AV) blocks secondary to hypothyroidism have also been reported [2-6]. We present a case of severe hypothyroidism causing symptomatic bradycardia and a high-grade AV block which was successfully treated with thyroxine hormone replacement without requiring cardiac pacemaker placement.

## Case presentation

**Figure 1:**
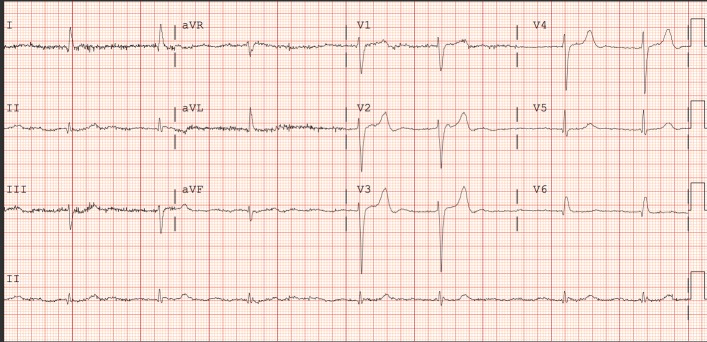
EKG on admission showing atrial fibrillation with slow ventricular rate EKG: Electrocardiogram

An 87-year-old man with a past medical history of hypertension and hypothyroidism was told by his cardiologist to present to the Emergency Department (ED) after he was found to have abnormal electrocardiogram (EKG) findings on the Holter monitor. He was seen by his primary doctor two days prior to presentation for arm pain and the EKG at that time showed an irregular rhythm for which the patient was referred to a cardiologist who placed a Holter monitor. The patient denied chest pain, palpitations, shortness of breath, or headaches at the time of presentation to the ED. Physical examination findings were as follows: pulse 36 beats/min, respiratory rate 16/minute, blood pressure 180/70 mmHg, percentage oxygen saturation was 96% on room air. EKG showed new-onset atrial fibrillation with slow ventricular response, frequent episodes of bradycardia to less than 40 beats per minute (bpm), and left bundle branch block (LBBB) as shown in Figure 1.

**Figure 2:**
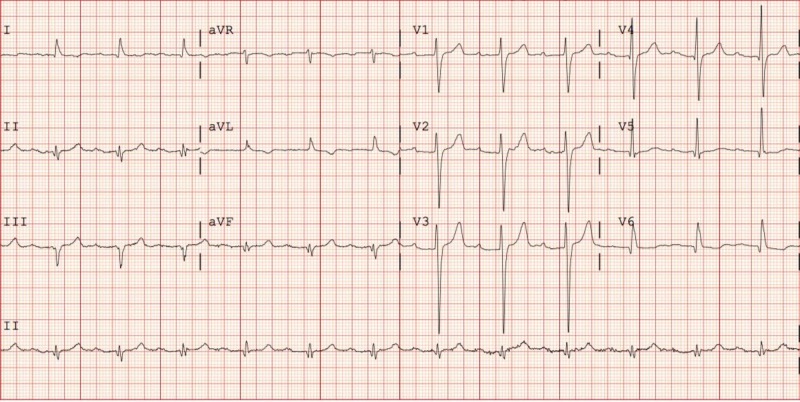
EKG two years before admission showing first degree AV block with LBBB EKG: electrocardiogram; AV: atrioventricular; LBBB: left bundle branch block

The patient was not taking any AV nodal blocking agents such as beta blockers, calcium channel blockers, or digoxin that could explain the cause of the slow ventricular rate. Initial labs showed normal pH and an unremarkable basic metabolic panel; complete blood count was within normal limits for age and gender. Thyroid stimulating hormone (TSH) was elevated to 74.03 IU/ml (reference range 0.39-4.08 IU/ml), with free thyroxine (fT4) decreased to 0.53 mg/dl (0.58-1.64 mg/dl). The patient reported that he was on levothyroxine 25 mcg daily for at least three years and he had been taking it on an empty stomach, one hour before breakfast daily. Baseline EKG two years ago showed LBBB with first degree AV block as shown in Figure 2.

The patient had a CHA2DS2-VASc score of three and was planned to be started on apixaban for prevention of thromboembolism. It was decided to hold off on rate control therapy for atrial fibrillation due to frequent episodes of bradycardia to less than 40 bpm. Echocardiogram showed ejection fraction (EF) of 46%-50%, mild diastolic dysfunction, and increased pericardial fat.

**Figure 3:**
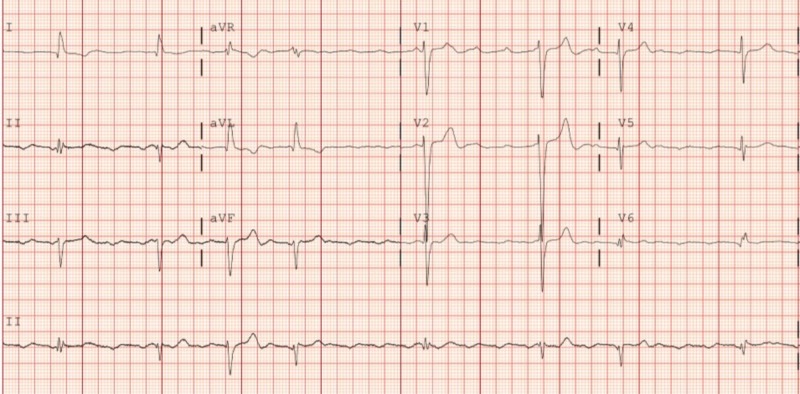
Atrial tachycardia with a variable high-grade AV block AV: atrioventricular

The patient was admitted to the telemetry unit, and the levothyroxine dose was increased to 50 mcg per oral daily. A repeat EKG the next day showed atrial tachycardia with a variable high-grade AV block as shown in Figure 3.

The patient also reported symptoms of dizziness with minimal exertion, and it was planned to insert a pacemaker for a high-grade AV block and symptomatic bradycardia. However, a subsequent telemetry review showed complete resolution of the high-degree AV block and pauses were no longer seen four days after increasing the dose of levothyroxine. Permanent pacemaker insertion was avoided, and the patient was discharged on levothyroxine 50 mcg daily, apixaban 5 mg twice daily for new-onset atrial fibrillation, and was advised to follow-up with a cardiologist for repeat thyroid function testing in four to six weeks.

## Discussion

Thyroid hormone is essential for life. It exerts its cardiovascular effects by binding to intracellular thyroid receptors (TR) which, in turn, bind to thyroid hormone response elements (TRE) on various genes and can affect gene transcription and, subsequently, protein translation. The thyroid hormone increases the basal metabolic rate in almost every tissue and organ system in the body by regulating gene expression, and the increased metabolic demands lead to changes in cardiac output, systemic vascular resistance (SVR), and blood pressure. In hypothyroidism, endothelial cell dysfunction and impaired vascular smooth muscle relaxation lead to increased SVR. These effects lead to diastolic hypertension. Thyroid hormone replacement therapy can restore endothelial-derived vasorelaxation and blood pressure to normal in most cases. Cardiac dysrhythmias have been reported with hyper as well as hypothyroidism. Bradyarrhythmias are typically associated with hypothyroidism [1].

A case reported published in 2008 presented a patient who was admitted to the hospital after a syncopal episode. The patient had a third-degree AV block secondary to severe hypothyroidism with a thyroid-stimulating hormone (TSH) level above 100 IU/ml. The patient required temporary pacing for a few days and was given treatment with levothyroxine. His heart block completely resolved after a few days, and the temporary pacing was subsequently terminated [2].

Kazim et al. conducted a study on AV blocks in patients with thyroid disease. A subgroup analysis of the study shows that seven out of 29 patients (24%) who had hypothyroidism and AV blocks had complete resolution of AV blocks after treatment with levothyroxine. However, the sample size was too small to draw any definitive conclusion [3]. Several patients with myxedema secondary to severe hypothyroidism and high-degree AV blocks had a complete reversal of AV blocks after treatment with thyroid hormone replacement for a few weeks and did not require cardiac pacing [4-6].

Most studies consider AV blocks due to hypothyroidism to be reversible; however, the literature is controversial and a few studies showed that most patients with AV blocks need permanent pacemaker placement. American College of Cardiology Foundation / American Heart Association / Heart Rhythm Society (ACC/AHA/HRS) 2008 guidelines for device-based therapy of cardiac rhythm abnormalities recommend permanent pacemaker implantation in patients with advanced second-degree and third-degree AV blocks who have symptoms (Class I recommendation, level of evidence C). However, the guidelines also give a Class III recommendation in favor of deferring pacemaker placement in patients who are asymptomatic and have a benign reversible cause of AV blocks such as Lyme disease, drug toxicity, or transient increases in vagal tone (level of evidence: B). There are no clear guidelines regarding how to manage patients with high degree AV blocks with severe hypothyroidism, and there is controversy in the literature.

Our patient had a baseline first-degree AV block and was found to be in new onset atrial fibrillation with slow ventricular response and a high-degree AV block. The AV block improved with supplementation of thyroxine. The decision to place a permanent pacemaker in patients with hypothyroidism should be individualized based on symptoms, comorbid conditions, and response to thyroxine replacement. A temporary pacemaker can be placed in select cases that require cardiac pacing due to the severity of symptoms or hemodynamic compromise, while awaiting restoration of normal sinus rhythm with thyroid hormone replacement.

## Conclusions

Advanced second and third-degree AV blocks with symptoms are considered to be an indication for pacemaker placement as per the latest guidelines by the AHA/ACC. However, the decision to insert a permanent pacemaker should be individualized, especially in patients with a reversible cause of heart block such as hypothyroidism. In our patient, there was complete resolution of the high-grade AV block within days after increasing the dose of his levothyroxine. Further studies may show better insight into the role of a permanent pacemaker in AV blocks in patients with thyroid dysfunction.
